# Colonization of Potato Rhizosphere by GFP-Tagged *Bacillus subtilis* MB73/2, *Pseudomonas* sp. P482 and *Ochrobactrum* sp. A44 Shown on Large Sections of Roots Using Enrichment Sample Preparation and Confocal Laser Scanning Microscopy

**DOI:** 10.3390/s121217608

**Published:** 2012-12-18

**Authors:** Dorota Krzyzanowska, Michal Obuchowski, Mariusz Bikowski, Michal Rychlowski, Sylwia Jafra

**Affiliations:** 1 Laboratory of Biological Plant Protection, Intercollegiate Faculty of Biotechnology UG&MUG, University of Gdansk, Kladki 24, 80-822 Gdansk, Poland; E-Mail: dorota.krzyzanowska@biotech.ug.edu.pl; 2 Laboratory of Molecular Bacteriology, Intercollegiate Faculty of Biotechnology UG&MUG, Medical University of Gdansk, 80-822 Gdansk, Poland; E-Mails: obuchowk@biotech.ug.edu.pl (M.O.); marbik8@wp.pl (M.B.); 3 Laboratory of Virus Molecular Biology, Intercollegiate Faculty of Biotechnology UG&MUG, University of Gdansk, Kladki 24, 80-822 Gdansk, Poland; E-Mail: michalr@biotech.ug.edu.pl

**Keywords:** bacteria visualization, fluorescent protein, PGPR, plant-associated bacteria, soil environment, transformation

## Abstract

The ability to colonize the host plants’ rhizospheres is a crucial feature to study in the case of Plant Growth Promoting Rhizobacteria (PGPRs) with potential agricultural applications. In this work, we have created GFP-tagged derivatives of three candidate PGPRs: *Bacillus subtilis* MB73/2, *Pseudomonas* sp. P482 and *Ochrobactrum* sp. A44. The presence of these strains in the rhizosphere of soil-grown potato (*Solanum tuberosum* L.) was detected with a classical fluorescence microscope and a confocal laser scanning microscope (CLSM). In this work, we have used a broad-field-of-view CLMS device, dedicated to *in vivo* analysis of macroscopic objects, equipped with an automated optical zoom system and tunable excitation and detection spectra. We show that features of this type of CLSM microscopes make them particularly well suited to study root colonization by microorganisms. To facilitate the detection of small and scattered bacterial populations, we have developed a fast and user-friendly enrichment method for root sample preparation. The described method, thanks to the *in situ* formation of mini-colonies, enables visualization of bacterial colonization sites on large root fragments. This approach can be easily modified to study colonization patterns of other fluorescently tagged strains. Additionally, dilution plating of the root extracts was performed to estimate the cell number of MB73/2, P482 and A44 in the rhizosphere of the inoculated plants.

## 1. Introduction

The plant rhizosphere supports a large microbial population by providing it with nutrients present in the root exudates [1]. In the resident microflora of the rhizosphere, harmless or beneficial commensals can be found, but also plant pathogens. The commensals may provide the roots with a basal level of protection against pathogens simply by occupying the niche [2]. Some of the strains however, known as plant growth-promoting rhizobacteria (PGPR), exhibit additional traits that can directly or indirectly benefit health and yield of the plant. Direct growth stimulation can be mediated by facilitated acquisition of nutrients, mainly phosphorus and nitrogen, or by modulation of the phytohormone equilibrium [2]. The indirect effect stems from increased plant resistance to diseases. This can result either from induction of the defense system of the plant or from antagonism shown by PGRPs towards plant pathogens (for review see [3,4]).

Since the first reports on plant beneficial microbes [5], extensive research has been conducted on their potential to act as bio-fertilizers and/or biological control agents (BCAs). Application of such microorganisms in agriculture is expected to reduce the use of chemical fertilizers and pesticides. Candidate PGPRs are usually obtained by screening large collections of bacterial isolates seeking biochemical or genetic traits known to, or assumed to be involved in plant beneficial activity (for a review see [6]). However, to be effective *in planta*, the candidate strains need to be able to establish and maintain a sufficient population in the host plant [7]. A variety of cell surface molecules contribute to the colonization process, as does efficient utilization of the major carbon and nitrogen sources available in the root exudates of a certain plant species or even a certain cultivar. Plant growth conditions, including soil type and occurrence of stress factors, also play a part in determining the composition of plant microflora. This complex web of interdependence makes it problematic to study and predict plant-microbe interactions, especially in case of soil-grown plants. Therefore, the colonization potential of each PGRP candidate needs to be experimentally determined.

Tracking the presence of such bacterial strain in an overcrowded environment as the rhizosphere requires selective detection methods. This can be accomplished after modification of the strain to obtain additional antibiotic resistance for selective growth and/or introduction of a reporter gene, such as a fluorescent protein-coding gene. Quantitative techniques for colonization study, providing cell count in colony forming units (CFU) per gram, cm or sq cm of root, include serial dilution plating on selective media and flow cytometry [8]. To visually demonstrate colonization and obtain qualitative data, such as the location of bacteria on plant roots, classical fluorescence and confocal laser scanning microscopy (CLSM) can be applied [9]. CLSM is a powerful investigation tool for studying many aspects of root physiology [10], including interaction of the plant with rhizobacteria and fungi [9,11]. In combination with fluorescently-tagged microorganisms, the method can provide 3D images of the studied objects with minimum invasive manipulation. Noteworthy, apart from modified strains, immunochemistry and fluorescent *in situ* hybridization (FISH) can also be used for specific staining. However, due to the problem of cross-reactivity, labeled antibodies are often used in combination with gnotobiotic plant growth conditions [12] and FISH is a golden standard when it is necessary to localize organisms representing different taxonomic ranks in one sample [13].

The aim of this study was to investigate the potential of three candidate PGPRs—*Bacillus subtilis* MB73/2, *Ochrobactrum* sp. A44 and *Pseudomonas* sp. P482—to colonize the rhizosphere of potato. Fluorescence microscopy and CLSM were employed, as well as the quantitative dilution plating approach. For the purpose of microscopy, root samples were embedded in an enrichment medium, triggering *in situ* growth of mini-colonies. The developed method enables visualization of bacterial colonization sites on large root fragments.

## 2. Experimental Section

### 2.1. Bacterial Strains, Plasmids and Culture Conditions

All bacterial strains and plasmids used in the present study are listed in Table 1. Bacterial cells were grown on Miller’s LB agar plates (Novagen, Madison, WI, USA) at 28 °C. When necessary, the medium was supplemented with antibiotics at the following concentrations: gentamicin 20 μg·mL^−1^, ampicillin 50 μg·mL^−1^, spectinomycin 100 μg·mL^−1^, cycloheximide 200 μg·mL^−1^ and rifampicin 100 μg·mL^−1^. For each experiment, bacteria were freshly plated from deep-frozen (−80 °C) glycerol stocks.

### 2.2. Introducing *gfp* Reporter Cassette into the Genome of *B. subtilis* MB 73/2

Insertion of a *gfp* reporter cassette into the *amyE* locus in the genome of *B. subtilis* MB 73/2 was performed by transformation of chromosomal DNA obtained from *B. subtilis* OMG982 into the recipient strain and homologous recombination. Protoplast electroporation method, described by Romero *et al.*[19], was applied to introduce DNA into the *B. subtilis* MB 73/2 cells.

### 2.3. Introducing Plasmid-Born *gfp* Reporter into *Ochrobactrum* sp. A44 *and Pseudomonas* sp. P482

To obtain the GFP-expressing derivatives of two Gram negative strains, *Pseudomonas* sp. P482 and *Ochrobactrum* sp. A44, bacterial cells were transformed with pPROBE-GTkan [18]. The vector was introduced into the cells by electroporation with the Gene Pulser Xcell (Bio-Rad, Hercules, CA, USA). Cells from an overnight culture in LB medium were harvested by centrifugation and washed twice with cold 10% glycerol. Two microliters of pDNA (120 ng·μL^−1^) were added to a 100 μL aliquot of competent cells, and placed in a 0.2 cm electroporation cuvette. The following pulse conditions were applied: 12.5 kV·cm^−1^, 25 µF, 200 Ω for the *Pseudomonas* sp. P482 [15,20] and 15 kV·cm^−1^, 25 µF, 200 Ω for the *Ochrobactrum* sp. A44 [17,21].

### 2.4. Bacterization of the Potato Seed Tubers and Plant Growth Conditions

Certified potato seed tubers cultivar (cv.) Krasa, caliber 35–45 mm, were obtained from the Plant Breeding and Acclimatization Institute (Instytut Hodowli i Aklimatyzacji Roślin—IHAR, Bonin, Poland). Bacterial cells from an overnight culture on LB agar plates, grown at 28 °C, were harvested and suspended in 1% carboxymethylcellulose (Calbiochem, Merck KGaA, Germany) to obtain an optical density of 6 units in the McFarland scale (approx. log 8 CFU·mL^−1^, determined by plating). Carboxymethylcellulose was added to the inoculation mixture to enhance the adhesion of bacterial cells to the tubers, as previously described by Geels *et al.*[22]. The tubers were coated with the suspension and potted in 12 cm diameter pots filled with potato field-derived soil (source: Lake Golubie area, Kaszuby, Poland; soil type: very light soil, granulometric group: sand). Tubers coated with bacteria-free 1% carboxymethylcellulose were potted to serve as control samples. For extraction and dilution plating, the plants were grown for three weeks. Growth conditions were as follows: temperature 21 ± 2 °C, humidity 80 ± 5%, white light (Cool White, model TLD 58W/84o, photosynthetic photon flux 20 μM·s^−1^·m^−2^; Koninklijke Philips Electronics N.V., Eindhoven, The Netherlands) and a long day photoperiod (16 h light/8 h dark). For the microscopic analysis, four-week old plants were used, reaching potato growth stage II (vegetative growth) to early III (tuber initiation).

### 2.5. Sample Preparation for Microscopy

Plants were removed from the pots and excess soil was removed by shaking. Root samples (sections from stolon to the root tip) were cut, soaked in sterilized ddH_2_O and placed in sterile plastic Petri dishes. Enrichment medium, cooled down to 42–45 °C, was gently poured over the sample to embed the roots. The base of the enrichment medium was composed of: 2 g·L^−1^KH_2_PO_4_, 4.66 g·L^−1^K_2_HPO_4_, 1.33 g·L^−1^ (NH_4_)_2_SO_4_, 50 mg FeSO_4_, 80 mg MgSO_4_, 10 g·L^−1^agar, pH 7. For M73/2 GFP, spectinomycin (100 μM·mL^−1^) and glycerol (0.4%) were added. For A44 GFP and P482 GFP, the medium was supplemented with gentamicin (20 μg·mL−1) and glucose (0.4%). After settling, the samples were incubated at 28 °C for 36 h. When visualization of plant tissue was difficult using autofluorescence alone, the embedded roots were sprayed with 1 mg·mL^−1^ water solution of Fluoresent Brightner 28 (also known as calcofluor) (Sigma-Aldrich, St. Louis, MO, USA), 20 min prior to sample analysis.

### 2.6. Fluorescence and Confocal Laser Scanning Microscopy

Root preparations were analyzed without removing the roots from the surrounding medium (directly in the Petri dish). For initial visualization of bacterial mini-colonies on the roots, classical fluorescence microscopy analysis was performed with the Nikon Eclipse TE 300 inverted microscope (Nikon, Tokyo, Japan), equipped with a GFP-suitable filter system (excitation filter: 450–490 nm, barrier filter: 520 nm). Images were collected using a high resolution color digital camera (Hammamatsu Photonics C4742-95, Hammamatsu, Japan) and processed with LUCIA Image Software. The 10× objective was used. Due to the limitations of classical fluorescence microscopy when it comes to observing thick objects, it is difficult to obtain a sharp image of the whole root section. Thus, the images of the adjacent root surfaces were juxtaposed manually using graphic-processing programs (Corel Photo Paint and Microsoft Power Point). The confocal images were acquired using a Leica TCS LSI Macro Confocal (Leica Microsystems, Wetzlar, Germany). This CLSM device, dedicated to the analysis of macroscopic objects, is equipped with a 5× macro-objective, automated optical zoom system and tunable excitation and detection spectra. Leica Application Suite software was used for confocal images processing. This software enables automatic assembly of mosaic images from multiple adjacent fields of view.

### 2.7. Estimation of Bacterial Cell Number by Dilution Plating

Rifampicin-resistant clones of all tested strains were selected under antibiotic pressure [23]. To confirm their identity, REP-PCR was performed as described by Versalovic *et al.*[24] and the profiles obtained for the mutants were compared with the ones for the wild type (*wt*) strains. Potato seed tubers were inoculated with A44 Rif, P482 Rif and MB73/2 Rif and potted (see Section 2.4 for details). Three weeks after, root samples were collected (1–2 g), placed in extraction bags (Bioreba) and weighed. Sterile saline (0.9% NaCl) was added to the each sample-containing bag in 1:9 (w/v) ratio and the material was homogenized. Serial dilutions of the suspension were plated on LB agar supplemented with cycloheximide (200 μg·mL^−1^) and rifampicin (100 μg·mL^−1^). Following 48 h of incubation at 28 °C, bacterial colonies were counted and the mean value of CFU per gram of root fresh weight (CFU·gfw^−1^) was determined. A total of 6–8 plants were used *per* treatment in two replicates.

## 3. Results and Discussion

*Bacillus subtilis* MB73/2, *Ochrobactrum* sp. A44 and *Pseudomonas* sp. P482 isolates obtained from different plant-associated environments (Table 1) [15,16] showed antagonism towards pectynolytic bacteria of genera *Dickeya* and *Pectobacterium*, important pathogens of potato [25]. P482 and MB 73/2 inhibit the growth of the pathogens by production and secretion of the unidentified antibiotic compounds, whereas A44 disturbs quorum sensing-dependant regulation of virulence factors production by the pathogens cells [16,17]. For this study, inoculation of tubers was chosen since seed tuber treatment is broadly used in potato production to prevent seed-borne and soil-borne diseases.

To selectively re-isolate the A44, P482 and MB73/2 cells from the rhizosphere and to estimate their number, a classical microbiology approach was applied, involving the use of spontaneous rifampicin resistant mutants and dilution plating. Potato root samples (cv. Krasa) were analyzed three weeks after tuber inoculation and potting. All three strains were recovered from the root samples. The following cell numbers were detected: A44 Rif log 4.29 ± 0.58 CFU·gfw^−1^, P482 Rif log 3.75 ± 0.29 CFU·gfw^−1^, MB 73/2 Rif log 3.2 ± 0.08 CFU·gfw^−1^. Population size of an introduced PGPR usually drops rapidly with time [26]. With regard to the time of sampling, we could consider A44 an efficient potato root colonizer and P482 and MB73/2 as moderate to weak ones. This is, however, rather arbitrary. Despite the extensive research conducted on the biology of PGPRs, the concept of ‘efficient colonization’ is not uniformly defined. The studied plant-microbe systems, as well as the experimental setups employed by different research groups, often vary to such an extent that the results cannot be compared. Apart from the specificity of each plant-microbe system, the following variables can be distinguished: the size of the initial inoculum, the inoculation method, the conditions of plant growth, the time of sample harvesting post inoculation and the unit adopted to express the results (CFU·gfw^−1^, CFU·cm^−1^ or CFU·sq·cm^−1^). Probably the best way to define ‘efficient colonization’ is to judge it by the lack or presence of a desired effect that colonization has on the host plant (if known). The efficient colonization ‘threshold’ can differ depending on the effect of interest (*i.e.*, induction of systemic resistance, competition with pathogens, bioremediation of toxic compounds).

The main aim of this work was to visually demonstrate the presence of A44, P482 and MB73/2 on the roots of soil-grown potato plants. For this purpose, fluorescence and CLSM were employed. To provide the fluorescence signal, tested strains were GFP-tagged. The Gram negative *Ochrobactrum* sp. A44 and *Pseudomonas* sp. P482 were labeled with *gfp* borne on the pPROBE-GTkan vector under a constitutive *nptII* promoter [18]. The pVS1/p15a *ori* present on the plasmid enables its stable propagation in bacterial cells without selection [27]. Plasmids from the pPROBE series were already successfully applied for tagging bacterial cells for flow cytometry and microscopy (*i.e.*, [8,27,28]).

Cells of *B. subtilis* MB73/2 proved to be very difficult to transform. This is not an uncommon problem. Environmental isolates are more problematic to genetically modify than the laboratory strains, especially when it comes to Gram positive bacteria. To overcome this obstacle, we have applied the protoplast electroporation method, described previously by Romero *et al.*[19]. It enabled us to introduce the λ_R_-*gfp* fusion from OMG 982 [14] into the *amyE* locus of the MB73/2 genome and to obtain constitutive GFP expression in the modified cells.

**Figure 1.**
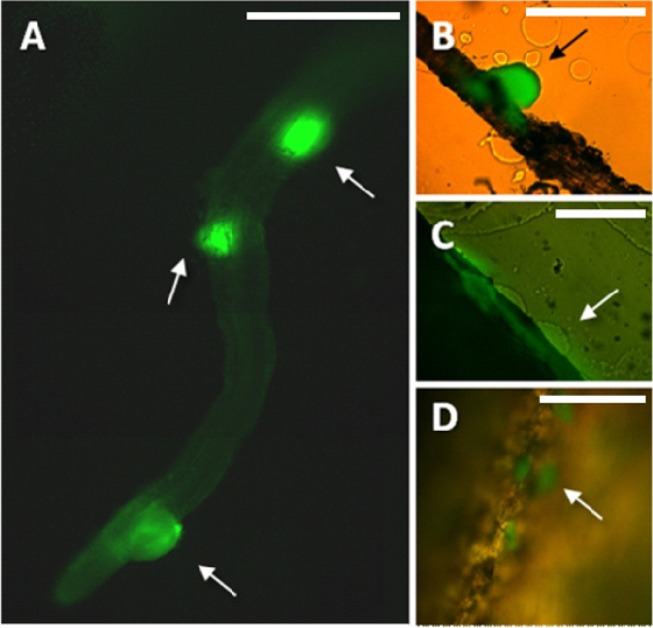
Mini-colonies of GFP-expressing strain ***B. subtilis*** MB73/2 GFP. Bacteria could be detected on potato roots (**A**), stolon and juvenile progeny tuber ((**B**) and (**C**) respectively), and the peel of the mother tuber (**D**). Images were obtained with Nikon Eclipse TE 300 fluorescence microscope. Figure A was created by manual juxtaposition of 3 images. Arrows indicate the position of MB73/2 GFP mini-colonies. Scale bar in (**A**–**D**), 500 μm.

Preparation of samples for microscopic analysis involved embedding of the harvested roots in a specially formulated enrichment medium. Enrichment was already proved useful for showing bacterial infection of plant tissue [29]. In this study, the composition of the medium enabled *in situ* growth of mini-colonies of the studied PGPR candidate while inhibiting the growth of most other microorganisms. The samples were not removed from the embedding matrix for microscopic observation, in this way enabling preservation of the original colonization sites. Enrichment approach applied in this study enables fast screening for bacterial colonization sites along the roots even in case of sparse target microbial populations. It is important as certain strains can prefer to colonize different segments along the root (*i.e.*, the root base or the root tip) [30]. Using this method, in combination with fluorescence microscopy, we have found that the small population of MB73/2 on potato root is non-uniformly distributed: neighboring colonization sites could be observed (Figure 1(A)) as well as large unoccupied areas. M73/2 GFP cells were detected both in the root maturation zone and at the root base. Interestingly, the cells were more abundant on the stolon and the peel of immature progeny tubers than they were on the root (Figure 1(B,C)).

Even small plant roots are thick when observed under a microscope. Thus, it is convenient to use CLSM to analyze these types of preparations, as it allows 3D visualization of the studied structures. A disadvantage of CLSM when comparing to fluorescence microscopy is long scanning time in order to obtain the desired images, especially when multiple scanning of each layer (averaging) is applied for higher quality. An advantage of the sample preparation method presented in this study is that it does not involve removing of the roots from the enrichment matrix. Thus, there is no risk of preparation damage and drying even when long-time or repeated scanning is required.

A common problem when analyzing plant tissue samples with GFP-tagged derivatives is the presence of many other light-absorbing pigments [31]. These are often fluorophores which produce high intensity background autofluorescence therefore making it difficult to separate their output from that emitted by the fluorescent probes [32]. This problem is amplified especially when low-intensity signal detection is required close to meristems or healing tissue. A confocal microscope equipped with a spectral detector is a valuable tool for overcoming this inconvenience.

**Figure 2.**
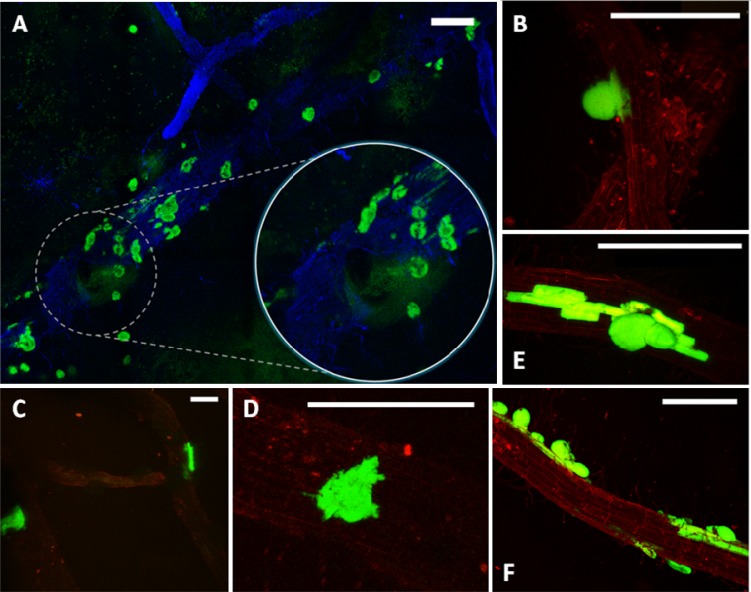
Colonization of potato roots by three different GFP-tagged bacterial strains. Images were obtained with Leica TCS LSI Macro Confocal. Green pseudocolor indicates the position of GFP-expressing mini-colonies. ((**A**), (**B**)) *Ochrobactrum* sp. A44 GFP. Figure A is a result of subsequent scanning of 6 fields of view, followed by automatic juxtaposition of the images. The preparation in figure A was stained with Fluorescent Brightner 28; ((**C**),(**D**)) *B. subtilis* MB73/2; (**E**, **F**) *Pseudomonas* sp. P482. Scale bar in (**A–F**), 500 μm.

Considering the challenges and expectations regarding our type of samples, we have employed the Leica TCS LSI Macro Confocal. This microscope represents a group of modern CLSM devices designed for *in vivo* imaging, *i.e.*, in developmental biology. The 5× macro-objective of the Leica TCS LSI provided a large field of view, suitable for visualizing large sections of roots. When necessary, the continuously adjusted optical zoom assured easy switching from overview to details (single mini-colonies). The 488 nm laser line was used for excitation of the GFP and the signal was detected in a 500–525 nm emission band. To obtain the ‘root context’ for mini-colonies, the plant tissue autofluorescence was induced by a 532 nm laser line and detected in a broad emission band (>590 nm). The tunable excitation and detection spectra allowed a clear separation of the plant autofluorescence from the reporter protein signal. Similar microscope settings were successfully used by Lagopodi *et al.* to show GFP-tagged fungal hyphae on tomato roots [11]. In case that the autofluorescence is too weak and signal enhancement is necessary, staining can be applied to visualize plant tissue. Here, we have tested the applicability of Fluorescent Brightner 28 (Figure 2(A)), induced with a 405 nm laser line, although other stains can also be used, *i.e*., propidiumiodide [33].

The employed enrichment method, by triggering in situ growth of mini-colonies, enabled visualization of bacterial colonization sites on relatively large root fragments (Figures 1(A) and 2(A)). As a point of reference, magnifications used in other colonization studies are typically five to ten times greater, resulting in a smaller field of view (*i.e.*, [34]). The few exceptions concern observation of high-density bacterial populations, forming relatively large bacterial patches on the root surface [9]. The enrichment approach removes the limitations encountered when tracking individual bacterial cells on a relatively large surface area of the root, making it a helpful tool in studying colonization pattern of small and scattered bacterial populations.

The method described in this study can be easily applied to other bacterial strains by adjusting the composition of the enrichment medium. Regarding the possibilities offered by the modern CLSM devices (multiple fluorescence channels available), one could modify the method described above to serve co-colonization study. This would require tagging the second strain with a different fluorescent protein reporter in concert with the same plasmid- or genome-borne antibiotic resistance marker for both strains.

## 4. Conclusions

In this study, we have evaluated the potential of three candidate PGPRs—*B. subtilis* MB73/2, *Ochrobactrum* sp. A44 and *Pseudomonas* sp. P482—to colonize the potato rhizosphere following seed tuber bacterization. Rifampicin-resistant mutants and dilution plating were used for a quantitative study. Regarding the inoculation conditions and the time of sampling, we can consider A44 an efficient root colonizer, and both P482 and MB 73/2 a moderate ones.

To detect bacterial colonization sites on the potato roots, we have created GFP-tagged derivatives of the tested strains and designed an enrichment sample preparation method for classical fluorescence microscopy and CLSM. Both methods proved suitable for observation of bacterial colonization sites. However, although fluorescence microscopy enables fast overview of the preparations, it is not very suitable to handle thick preparations (*i.e.*, roots). Obtaining a sharp image of a larger root section required manual juxtaposition of many adjacent fields of view. Confocal microscopes designed to observe large structures (*i.e.*, embryos, tissue fragments) have the advantage of a large field of view, uncommon in most confocal microscopes, dedicated to research at the cell level. Combining this feature with a spectral detector and a wide range of available fluorescent proteins, stains and probes makes it a powerful investigation tool for studying the physiology of plant roots. This also includes research on the ability of PGPRs with potential agricultural application to colonize the rhizosphere of the target plant species. The enrichment method described in this study enables *in situ* growth of mini-colonies on the root surface. This removes limitations resulting from the small size of bacterial cells in comparison to relatively big size of the plant root. In concert with wide field microscopy, the described approach enables fast visualization of bacterial colonization sites on large root fragments, even in case of small and scattered bacterial populations. Considering that the composition of the enrichment medium can be easily modified, the proposed method can also be used for selective enrichment and detection of other fluorescently-tagged bacterial strains.

## Figures and Tables

**Table 1.** Bacterial strains and plasmids used in this work.

**Strain/Plasmid**	**Relevant Features**	**Reference(s)**
*Bacillus subtilis* MB 73/2	Isolated form meadow soil in Zulawy area, Poland; produces an unidentified antibacterial compound with strong activity against *Dickeya* sp.	Bikowki, M., unpublished
MB 73/2 GFP	Strain *Bacillus subtilis* MB 73/2 carrying *gfp* under control of λ_R_ promoter in *amyE*, spectinomycin resistance cassette (Spc^R^); constitutive expression of *gfp*	This work
MB 73/2 Rif	Spontaneous rifampinin resistant mutant of the respective strain	This work
*B. subtilis* OMG 982	*gfp* under the control of λ_R_ promoter in *amyE*, spectinomycin resistance cassette (Spc^R^)	[14]
*Pseudomonas* sp. P482	Isolated from tomato rhizosphere (Gdansk, Poland); produces an unidentified antibacterial compound; decreases soft rot symptoms caused by *Pectobacteium* sp. on potato	[15]
P482 GFP	Respective strain carrying the pPROBE-GTkan vector	This work
P482 Rif	Spontaneous rifampinin resistant mutant of the respective strain	This work
*Ochrobactrum* sp. A44	Isolated from potato rhizosphere in the Netherlands; inactivates a broad spectrum of AHL-type signal molecules involved in *quorum sensing*	[16,17]
A44 GFP	Respective strain carrying the pPROBE-GTkan vector	This work
A44 Rif	Spontaneous rifampinin resistant mutant of the respective strain	This work
pPROBE-GTkan	A vector carrying *gfp* gene under the control of constitutive *nptII* promoter; pVS1/p15a *ori* of replication enables stable plasmid propagation without antibiotic selection, gentamicin resistance cassette (Gm^R^)	[18]
